# EEDC: An Energy Efficient Data Communication Scheme Based on New Routing Approach in Wireless Sensor Networks for Future IoT Applications

**DOI:** 10.3390/s23218839

**Published:** 2023-10-30

**Authors:** Divya Gupta, Shivani Wadhwa, Shalli Rani, Zahid Khan, Wadii Boulila

**Affiliations:** 1 Department of Computer Science and Engineering, Chandigarh University, Mohali 140413, India; divya1907gupta@gmail.com; 2 Chitkara Uiversity Institute of Engineering and Technology, Chitkara University, Punjab 140401, India; 3 Robotics and Internet-of-Things Laboratory, Prince Sultan University, Riyadh 12435, Saudi Arabia; zskhan@psu.edu.sa (Z.K.); wboulila@psu.edu.sa (W.B.); 4 RIADI Laboratory, National School of Computer Sciences, University of Manouba, Manouba 2010, Tunisia

**Keywords:** IoT applications, clustering, energy efficiency, hierarchical framework, WSN-IoT, future communication

## Abstract

Wireless Sensor Networks (WSNs) and the Internet of Things (IoT) have emerged as transforming technologies, bringing the potential to revolutionize a wide range of industries such as environmental monitoring, agriculture, manufacturing, smart health, home automation, wildlife monitoring, and surveillance. Population expansion, changes in the climate, and resource constraints all offer problems to modern IoT applications. To solve these issues, the integration of Wireless Sensor Networks (WSNs) and the Internet of Things (IoT) has come forth as a game-changing solution. For example, in agricultural environment, IoT-based WSN has been utilized to monitor yield conditions and automate agriculture precision through different sensors. These sensors are used in agriculture environments to boost productivity through intelligent agricultural decisions and to collect data on crop health, soil moisture, temperature monitoring, and irrigation. However, sensors have finite and non-rechargeable batteries, and memory capabilities, which might have a negative impact on network performance. When a network is distributed over a vast area, the performance of WSN-assisted IoT suffers. As a result, building a stable and energy-efficient routing infrastructure is quite challenging in order to extend network lifetime. To address energy-related issues in scalable WSN-IoT environments for future IoT applications, this research proposes EEDC: An Energy Efficient Data Communication scheme by utilizing “Region based Hierarchical Clustering for Efficient Routing (RHCER)”—a multi-tier clustering framework for energy-aware routing decisions. The sensors deployed for IoT application data collection acquire important data and select cluster heads based on a multi-criteria decision function. Further, to ensure efficient long-distance communication along with even load distribution across all network nodes, a subdivision technique was employed in each tier of the proposed framework. The proposed routing protocol aims to provide network load balancing and convert communicating over long distances into shortened multi-hop distance communications, hence enhancing network lifetime.The performance of EEDC is compared to that of some existing energy-efficient protocols for various parameters. The simulation results show that the suggested methodology reduces energy usage by almost 31% in sensor nodes and provides almost 38% improved packet drop ratio.

## 1. Introduction

The amount of energy stored in a node’s battery impacts the node’s lifespans [1,2]. The stored energy is used for a variety of node tasks, including sensing, gathering, analyzing, and communication. Sensor node batteries are typically tiny and cannot be changed or recharged. Although energy-harvesting systems exist, they cannot eliminate the necessity for energy management. As a result, the most difficult task is to organize the limited battery power by adopting energy-effective protocols for WSNs. WSNs are used for a variety of purposes, including military surveillance, remote patient monitoring in healthcare, measuring environmental temperature, humidity, light, pressure, monitoring machinery in industries, and so on [3,4,5]. WSN design difficulties include energy conservation, scalability, data aggregation and compression, security, routing, resource constraints, and so on. To overcome these design challenges, a multidisciplinary approach involving hardware design, networking protocols, algorithm development, and domain-specific knowledge is required. The goal of research in these areas is to produce robust and efficient solutions that allow for the effective deployment of wireless sensor networks across a wide range of Future IoT applications. Because sensor nodes are self-contained devices that rely entirely on their batteries to function, several academicians have recently been investigating energy-efficient clustering and routing strategies to reduce energy usage in sensor nodes.

The Internet of Things (IoT) and Wireless Sensor Networks (WSNs) are ideas that are closely linked and frequently overlap in their applications and technologies. The Internet of Things can be viewed as an evolution of WSNs [6]. As WSNs are critical to the Internet of Things (IoT), sensors in IoT-based WSNs continuously monitor the surroundings and promptly alert the base station (BS) whenever any incident is detected. An IoT-based WSN also includes a gateway for uploading acquired data to the IoT Cloud. In some setups, the BS might serve as the gateway. Users can access the data saved in the IoT Cloud remotely at any time. The incorporation of IoT principles into WSNs expands their capabilities and creates chances for novel applications across different areas. IoT with WSN support has brought about a number of applications and improved the convenience of everyday living. The distributed network across different WSN applications for the Internet of Things may be static or dynamic depending on its specific requirements [7,8]. Smart healthcare monitoring systems enable dynamic networks, unlike static networks, which are supported by crop monitoring and environmental monitoring, for instance. WSN-assisted IoT networks cover a larger area than WSN networks and have a higher number of nodes (due to resource constraints) compared to WSN networks. As a result, standard WSN methods may be ineffective in a scalable WSN-aided IoT network. Many researchers used the cluster-based hierarchical framework to solve the scalability and network lifetime problems [9,10]. The choice and management of Cluster Head (CH) nodes is the primary focus of clustering-based routing systems. The CH receives data from the cluster’s nodes. The CH is in charge of aggregating data from localized cluster participants and transmitting it to the base station or surrounding CHs towards the Base Station (BS). This adds additional strain to CH, leading to more energy consumption as compared with ordinary nodes. Moreover, the CH towards BS also serves as Relay Nodes (RN) for the transmitting packets received from far located CHs, and therefore, depletes their energy more faster [11,12]. To address energy-related issues in scalable WSN-IoT environment, this research aims to design an Energy Efficient Data Communication (EEDC) scheme by utilizing a multi-tier hierarchical clustering framework where each tier is further divided into various regions.The proposed routing protocol aims to provide network load balancing and convert communicating over long distances into shortened multi-hop distance communications, hence enhancing network lifetime. This paper’s main contribution can be summarized as follows:

- This work discusses Region-based Hierarchical Clustering for Efficient Routing (RHCER) that employs energy-efficient clustering and routing techniques. RHCER employs a novel CH selection mechanism to ensure efficient deployment for scalable networks.
- To ensure efficient long distance communication along with even load distribution across all network nodes, a subdivision technique was employed in each tier of the proposed framework.
- RHCER considers three critical parameters: distance from BS(D), threshold energy (TE), and signal-to-noise ratio (SNR) as a multi-criteria decision-making function f(n) for cluster head selection.
- In the findings section, the outcome of RHCER is compared to that of various existing energy-efficient protocols in the same area. According to the findings from simulations, the suggested model beats competitors in terms of minimizing energy consumption in sensors and prolonging the lifetime of WSN-associated IoT.

This article is divided into several sections: Section 2 discusses the prior work along with problem statement, Section 3 describes the System model along with required settings. Section 4 explains the proposed EEDC scheme in detail. Section 5 discusses the simulation environment and experimental results, and Section 6 concludes and discusses future work in this domain.

## 2. Related Work

Considering the huge and scalable networks, the difficulty in WSN-assisted IoT differs differently from that of regular wireless and wired networks. The usual routing protocol, which is based upon IP and does not provide scalability, is insufficient for the WSN-assisted IoT [13]. As a result, a wide variety of efficient routing methods have been created to satisfy the demands of WSN-assisted IoT [14,15]. The majority of routing protocols fall into one of three categories: data-centered routing, GPS-based routing, and hierarchical routing. The best routers in every cluster are promoted to the title of Cluster Head (CH) in hierarchical routing, which groups nodes into clusters. The local member transmits the data to the cluster’s CH. The data are combined by CH into one packet [16]. A number of researchers have developed various clustering strategies in order to increase the lifetime of networks and data transmission efficiency.

The work in [17] introduced the low energy adaptive cluster hierarchy (LEACH), with the goal of introducing the concept of a cluster-based strategy while improving energy efficiency over older systems. It is intended for use in applications where each sensor is restricted by power sources, such as batteries. LEACH divides sensor nodes into clusters, with each cluster having an assigned node known as the “Cluster Head” (CH). The CH is in charge of aggregating and transferring data from its cluster nodes to a central base station or sink node. LEACH’s fundamental idea is to spread energy consumption equitably across the network by rotating the CH duty among the sensor nodes over time. While LEACH was created to extend the life of wireless sensor networks, it does have several limitations that become more apparent when applied to bigger and more complicated contexts, such as IoT networks. As the network is expanded across a large area, the amount of distance between CH and BS grows, resulting in huge energy loss. Second, the LEACH CH selection process is randomized and does not guarantee the most suitable node allocation for CH.

Table 1 presents the comparison among various existing solutions in this domain.

Multi-hop LEACH (M-LEACH) is an expansion of the standard LEACH protocol that integrates multi-hop transmission to overcome the restrictions of traveling a long distance between Cluster Heads (CHs) and Base Stations (BSs) in wireless sensor networks (WSNs) [19]. M-LEACH attempts to increase energy economy and network performance by allowing intermediary nodes to operate as data relays. The primary idea underlying M-LEACH is to build a multi-hop link from the CHs to the BS, where data are transmitted across numerous intermediary nodes before arriving at the final destination. This helps to distribute the network’s energy load more equally and allows for communication over longer distances without severely depleting the energy of CHs.

To address the shortcomings of LEACH, a centralized form of LEACH (LEACH-C) was proposed. LEACH-Centralized combines the advantages of both LEACH and centralized techniques in order to deliver a more energy-efficient and controlled data collection process [18]. A centralized controller or base station is important in managing network functioning and data communication in LEACH-C. Unlike the original LEACH, cluster heads (CHs) gather and broadcast data autonomously.

The authors of [23] proposed Information Systems (PEGASIS), an upgraded version of LEACH. The network of nodes and sensor nodes uses PEGASIS to enable data transmission and reception from their nearby neighbors. One network node is chosen at random to serve as the leader node in each chain. Data are sent across the network by the leader node to the base station through all nodes’ communication with one another. Due to an even distribution of resource consumption, which leads to less loss of energy per round in the network, PEGASIS offers a longer lifetime than LEACH.

The PEGASIS protocol was altered by Jafri and his associates in [24] to incorporate the multi-head chain and the sink mobility idea. The sink is permitted to go along a predetermined path from one area to another. At the sojourn location (where the sink briefly resides for data collection), it waits for a sojourn time. Before the network is deployed, the sojourn location is established. A sink’s sojourn time is the period of time during which it remains at a predetermined location and gathers information from the chain leaders. Compared to PEGASIS, this strategy extends the network lifetime.

Another routing technique created for wireless sensor networks (WSNs) is the Hybrid Energy-Efficient Distributed Clustering (HEED). HEED uses a hybrid strategy that includes both centralized and distributed processes to balance energy consumption, extend network lifetime, and increase overall data collection efficiency in WSNs. HEED is especially efficient in circumstances with densely deployed sensor nodes because it takes advantage of node proximity to optimize cluster formation and communication.

The Energy-Efficient K-Means Technique (EKMT) [20] is based on the general concept of K-mean clustering with energy as an add-on parameter.The K-Means clustering algorithm is a prominent unsupervised machine learning technique for partitioning a dataset into a fixed number of groups (k). The center of each cluster is derived as the mean of the data points within that cluster. The algorithm distributes data points to clusters iteratively based on their closeness to cluster centers, and the centers are updated depending on the newly assigned points. An energy-efficient form of K-Means is constructed to reduce energy usage during the clustering process, particularly in resource-constrained contexts such as wireless sensor networks (WSNs). The proposed technique reduces communication distance between nodes while increasing network lifetime. However, in an unsecured and unlimited space environment, the proposed method is ineffective since it is vulnerable to intentional exploitation and may be damaging to sensor data.

Further, Improved Chain-Based Clustering Hierarchical Routing (ICCHR) works by organizing CHs in a sequential chain-like structure [21]. Each CH act as in charge of a subset of clusters, with data transmitted down the chain until it reaches a sink or base station. This chaining strategy brings benefits such as lower overhead and more efficient data aggregation. From the simulated outcomes, the suggested ICCHR algorithm reduces the energy consumption ratio while spreading the load uniformly among sensor nodes.

In the framework of Wireless Sensor Networks (WSN) for Internet of Things (IoT) applications, the study in [22] proposed Energy-Aware Cluster-based Routing (EACRLEACH). The selection of Cluster Heads (CH) is an important part of the clustering process in this protocol. This protocol likely aims to select Cluster Heads that have sufficient energy, good connectivity with neighboring nodes, proximity to the sink, and possibly an even distribution of CH roles throughout the network by combining Remaining Energy, Number of Neighbors, Distance between Sensor Node and Sink, and Number of Times Node Acts as CH.

The authors of [14] suggested a hierarchical routing technique for fog-assisted sensor networks. Regular sensor nodes and CHs just serve to forward data packets. Because fog nodes are superior in terms of storing, analyzing, and computation, they make all routing decisions.

We examined the aforementioned protocols and found a number of shortcomings with Cluster Head (CH) selection and organization that fall short of the fundamental needs of the WSN-assisted IoT network. To ensure connection to the network and data transmission, robustness is necessary [25,26]. However, the techniques outlined in this section might not be able to maintain network interaction because of the early demise of nodes close to BS. The objective of these routing protocols is energy optimization. They do not, however, consider additional WSN-assisted IoT features, such as scalability, load distribution, and other related ones. The goal of this research is to increase network lifetime by providing network load balancing and turning long-distance communications into shorter multi-hop distance communications.

## 3. System Model

This section provides a general description of the deployment framework for any smart IoT application. The subdivision technique, in order to split the network into regions and clusters, has been further discussed. Following that, several assumptions concerning communication flow inside network nodes have also provided.

### 3.1. Proposed Deployment Framework Architecture

**Figure 1:**
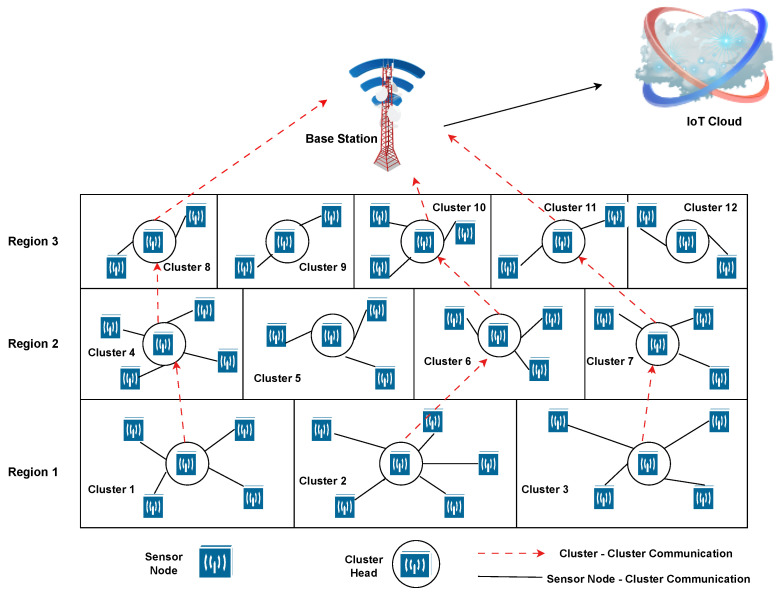
Proposed multi-tier clustering framework for WSN-IoT applications.

The proposed deployment of the multi-tier clustering framework for the smart WSN-IoT network is demonstrated in Figure 1. The framework considers a rectangular network area where all the nodes in the network are stationary and uniform. Each sensor node is equipped with GPS and a temperature sensor and they are randomly distributed across all network areas. Each node may communicate with the Base Station by varying its transmission range. The Base Station is present at the top in the uppermost layer and is responsible for collecting information from network nodes and sending it further to the IoT cloud for processing. The sensor nodes periodically send data to BS. The network is divided into various regions and each region is further divided into various sub-regions, which are commonly referred to as clusters. To ensure even load distribution across all network nodes, area covered by regions keep on decreasing towards BS and the number of sub-regions in each region keeps on increasing towards BS. As a result, the lowest region is the one with the maximum occupied area and the least number of sub-regions (clusters), while the highest region is with the least covered area and the maximum number of sub-regions. The motivation behind this is listed as the highest region. It not only sends aggregated data towards BS but also sends traffic coming from lower regions to BS, thereby utilizing more energy and network resources. This implies reduced intra-cluster communication distance in higher regions with increased number of clusters. Moreover, the sub-regions present in any particular region occupy the same area.

### 3.2. Subdivision Technique

To increase the network lifetime by evenly distributing the load across all network nodes, the network is divided into regions and sub-regions. Assuming *r* as the radius of sensor node, the diameter of the node sensing area was $D_{n}$. The maximum size rectangle that could be formed within the node sensing area has a diagonal $D_{rec}$ and side length as $s_{l}en$. The breadth of the entire network area is presented as $Bred_{net}$.

Therefore, the number of regions that could be formed are calculated as:

(1) $$Number_{reg}=\frac{Bred_{net}}{r}+1$$

From mathematics we know,

$$D_{n}=2*r\;\;\;\;\;\;and\;\;\;\;\;\;D_{rec}=s_{len}*\sqrt{2}$$

Here, the length of both $D_{n}$ and $D_{rec}$ are the same and therefore:

$$D_{n}=D_{rec}$$

This implies

$$2*r=s_{len}*\sqrt{2}$$

$$s_{len}=\frac{2*r}{sqrt2}$$

The area covered by the rectangle is

(2) $$s_{len}*s_{len}$$

$$\frac{2*r}{sqrt2}*\frac{2*r}{sqrt2}$$

$$2*r^{2}$$

Assuming m∗n as the dimension of the bottom region (region 1), respectively, the number of sub-regions (clusters) that could be formed in this area are:

(3) $$N_{clust_{k}}=\frac{m*n}{2*r^{2}}$$

Keeping in mind that the number of clusters at bottom regions (region 1) are minimal, the number of clusters in the next regions would be:

(4) $$N_{clust_{k}}=N_{clust_{k-1}}+1\;\;\;\;\;\;where\;\;\;\;\;\;1\leq k\leq Number_{reg}$$

The breadth of the regions are:

(5) $$breadth_{reg_{upp}}=\frac{r}{\sqrt{3}}$$

(6) $$breadth_{reg_{mid}}=\frac{bred_{net}-breadth_{reg_{upp}}}{Number_{reg}-1}$$

(7) $$breadth_{reg_{low}}=bred_{net}-\sum_{i=2}^{Number_{reg}}breadth_{reg_{i}}$$

Here, $breadth_{reg_{upp}}$ denotes the topmost region which is nearest to BS, $breadth_{reg_{low}}$ represents the lowest region, and $breadth_{reg_{mid}}$ represents the middle region. Any region beyond these mentioned can be formed and area can be calculated based on the above calculations.

## 4. Proposed Energy Efficient Data Communication Scheme: Region Based Hierarchical Clustering for Efficient Routing (RHCER)

The RHCER is a multi-tier clustering protocol for improving the network lifetime by achieving efficient balance between load and energy depletion across all network nodes. The Base Station (BS) is presented at the top of the network. Each node in the network sends their data to BS in multihop way. BS is aware of the entire network location and divides the whole network into various regions and sub-regions, as mentioned in Algorithm 1: Line 1–6. The proposed RHCER protocol deploys the multi-tier network for an agriculture scenario. The selection of CH in each cluster is done via BS using Algorithm 2: Cluster Head Selection. Immediately after the selection of CH in each cluster, the local cluster nodes send Join-Req to CH. Upon the reception of the Join-Req message from the sensor nodes, CH sends a reply via an acknowledgment message. Once connection between sensor nodes and CH within a local cluster is established, sensor nodes start sending their sensed data to CH. CH aggregates the data received from the nodes and transmits the data to the nearest neighbor CH present in its upper region. The selection of nearest neighbor CH in the upper region (One region above the current region towards BS) is done through Algorithm 1: Line 13–22. In this process, CH is chosen based on the ratio of residual energy of CH of the upper region to the distance between CHs of local region and the upper region. The process repeats itself until the packets finally reach BS. The energy of selected CH periodically gets checked and compared with the energy threshold. If at any point in time the CH energy is found to be lower than the threshold, the CH selection process gets started in that cluster. The time required for the evaluation of Algorithm 1 depends on the number of regions and the number of clusters in each region, and was therefore computed as $N_{regions}*N_{clusters}$.


**Algorithm 1:** Region based Hierarchical Clustering for Efficient Routing (RHCER)
**Initial**: Rectangular Network with dimension $Len_{net}$ and $Bred_{net}$ and *n* randomly
         distributed nodes

**Start Procedure**

1 Set of Nodes N={1,2,3,.....n}
2 BS divided the network into regions and sub-regions (Clusters) based on
    Equations (1)–(7)
3 Declare $Region_{id}=\left\{Reg_{1},Reg_{2},Reg_{3},......Reg_{h}\right\}$
4 Declare $Cluster_{id}=\left\{Clus_{1},Clus_{2},Clust_{3},......Clus_{p}\right\}$
5 Declare CH={}          A set containing Id of nodes chosen as cluster heads
6 Assign $Region_{id}$ and $Cluster_{id}$ to each region and cluster, respectively.
7 **for each** region with $Region_{id_{i}}$
8 set $Local-region=current-region_{id_{i}}$
9 **for each** Cluster with $clust_{id_{i}}$ with in local-region
10 set $local-cluster=current-cluster_{id_{j}}$
11 Send the packets from local cluster ordinary node to local cluster CH
12 **end for**
13 $CH_{a}$= Cluster Head of nearest neighbor cluster in higher region, i.e.
     $CH_{a}.Reg_{id}$= $local-cluster.Reg_{id}+1$
14 $CH_{b}$= Cluster Head of nearest neighbor cluster in higher region, i.e.
     $CH_{b}.Reg_{id}$= $local-cluster.Reg_{id}+1$
15 Compute distance of local cluster CH $\left(CH_{l}\right)$ with $CH_{a}$ and $CH_{b}$ respectively
16 $Dis_{l,a}$ = $\sqrt{{\left(l_{x}-a_{x}\right)}^{2}+{\left(l_{y}-a_{y}\right)}^{2}}$
17 $Dis_{l,b}$ = $\sqrt{{\left(l_{x}-b_{x}\right)}^{2}+{\left(l_{y}-b_{y}\right)}^{2}}$
18 **if** $\frac{CH_{a}.Energy}{Dis_{l,a}}$>$\frac{CH_{b}.Energy}{Dis_{l,b}}$
19 set $CH_{elec}=CH_{a}$
20 **else**
21 set $CH_{elec}=CH_{b}$
22 send the packet from $CH_{l}$ to $CH_{elec}$
23 Finally sent the packets to BS.
24 **end for**

**End Procedure**


**Algorithm 2:** Cluster Head Selection
**Input:** Local Region, Local Subregion (Cluster), S: Set of nodes within cluster
**Output:** Local Cluster Head $\left(CH_{l}\right)$

**Start Procedure**

1 Declare Base Station Position = $\left\{BS_{x},BS_{y}\right\}$
2 **for each** node s∈S
3 calculate distance of s from BS
4 $D_{BS,s}=\sqrt{{\left(BS_{x}-s_{x}\right)}^{2}+{\left(BS_{y}-s_{y}\right)}^{2}}$
5 $Total-Distance=Total-Distance+D_{BS,s}$
6 **end for**
7 $Avg-Distance=\frac{Total-Distance}{\left|S\right|}$
9 Calculate Energy Consumption (EC) of s for Transmission, sensing
    and Processing.
10 $EC_{s}=Energy_{tran}+G+D_{BS,s}$          Considering *G* is constant
    for sensing and processing
11 $Total-EC=Total-EC+EC_{s}$
13 $Avg-EC=\frac{Total-EC}{\left|S\right|}$
14 $Threshold_{energy}=\frac{Avg-EC}{Avg-Distance}$
15 **for each** node s∈S
16 **if** $s.residual_{energy}>Threshold_{energy}$
17 s may participate in CH election process
18 $CH_{s}=s.residual_{energy}*\frac{1}{D_{BS,s}}*\frac{1}{SNR_{BS,s}}$
19 **else**
20 s will act as normal node in cluster
21 **end if**
22 **end for**
23 For all the nodes in set S where $CH_{s}$ has been computed, arrange them in
     decreasing order of their $CH_{s}$ values
24 $CH_{l}$ = first node of array

**End Procedure**




The selection of the most appropriate node as CH within the local cluster is crucial. The node with the highest residual energy within the local cluster can provide uniform energy depletion for the entire network. The energy of each node within a local cluster is mainly utilized for transmission. The energy consumed for sensing and processing is minimal and is therefore considered as constant G (Algorithm 2: Line 10). The distance between BS and node is also one of the factors impacting energy depletion. Satisfying the condition that residual energy is higher than chosen threshold, the Cluster Head election process in local cluster for eligible node determines its residual energy, distance from BS, and Signal to Noise (SNR) ratio from BS. The efficient cluster head selection through the multi-criteria decision-making function, which includes distance from BS(D), threshold energy (TE), and signal-to-noise ratio (SNR), would have a significant impact on packet drop ratio as the high number of requests would be satisfied by cluster heads first due to aggregation of data from nodes of the same cluster. The reduced packet drop ratio will ultimately enhance the packet delivery ratio.

From the list of eligible nodes in the cluster head election process, the node with the highest value was selected as CH. The ratio between a node’s residual energy and the network’s overall energy was used to calculate the threshold energy regarding the ideal number of CH [27]. The time required for the evaluation of Algorithm 2 depends on the number of nodes in each sub-region, computed as S+S, i.e., 2S, ignoring constant number 2, implies *S*.

## 5. Evaluation and Performance

The simulation environment employed to evaluate the efficiency of our proposed technique is presented first in this section. Following that, we identify the performance criteria, which is crucial to compute the performance of any network clustering technique. Finally, we give an extensive analysis of our simulation results.

### 5.1. Simulation Environment

The simulation of the proposed multi-tier clustering framework for energy efficient routing was performed using MATLAB R2015a. The parameters used for evaluation has been listed in Table 2. The whole network is divided into four zones. The bottom-most zone has only one cluster, while the uppermost zone has four clusters. The upper zone aggregates the traffic from lower zones and hence traffic at zone near BS is higher compared to the lower zone. The efficiency of the proposed approach has been analyzed and results have been compared against state-of-the-art clustering techniques such as LEACH, M-LEACH, EACRP, and EEMAC.

### 5.2. Results and Discussion

The simulation outcome of our proposed approach has been compared against various network performance metrics. The metrics used in this evaluation mainly include network throughput, energy consumption, and number of alive nodes after each round [28,29]. The detailed discussion on the results retrieved for these metrics has been provided below.

#### 5.2.1. Network Throughput

**Figure 2:**
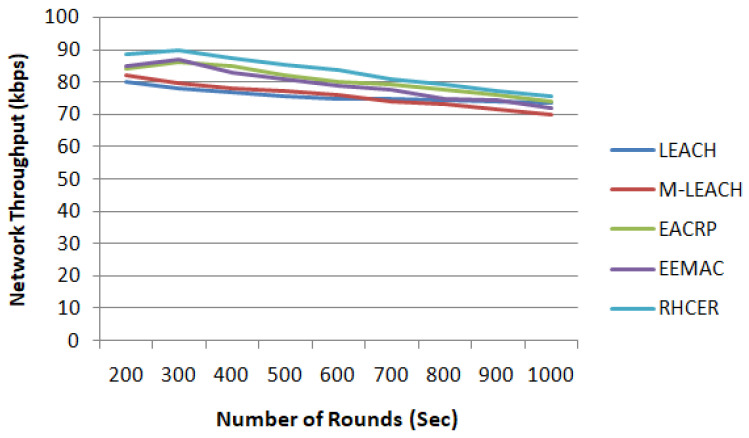
The impact on network throughput with varying number of simulation rounds.

The comparison of the proposed RHCER against considered state-of-the-art techniques in terms of network throughput under varied simulation rounds has been performed (refer Figure 2). The throughput is calculated as number of bits of data transmitted over network per sec. The simulation results show that the proposed scheme improves network throughput performance by 13%, 17.5%, 21%, and 26.8% for EEMAC, EACRP, M-LEACH, and LEACH, respectively. The improved network throughput is due to better network management in terms of regions and sub-regions, which leads to better load distribution. Moreover, unlike other considered solutions where signal strength has not been taken into account, the proposed scheme utilizes SNR for optimal selection of cluster heads, thereby improving the proportion of packets delivered in agricultural land due to their robust actions. In this regard, the suggested framework has a higher network throughput than previous alternatives.

#### 5.2.2. Energy Consumption

**Figure 3:**
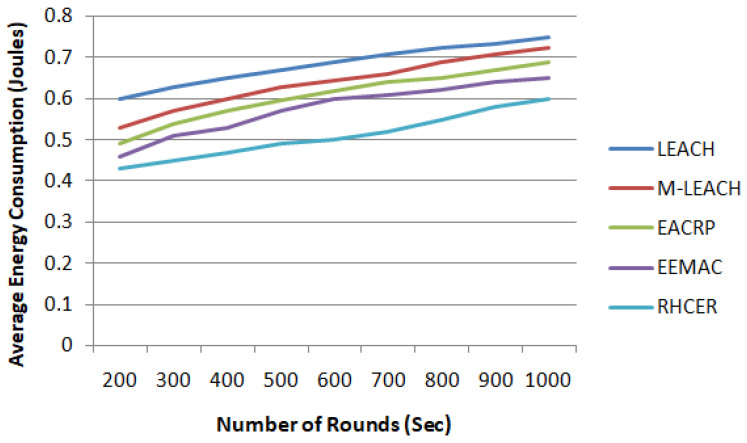
The impact on energy consumption with a varying number of simulation rounds.

The depletion of energy in the proposed scheme for the transmission of packet from sensor node to BS mainly includes energy used for transmission from sensor node to local CH, local CH to neighbor CH (number of rounds vary depending upon position of local zone in network), and finally from CH in the uppermost Zone to BS. The energy consumption for the proposed scheme in comparison to other solutions is analyzed in Figure 3.

The results revealed a better performance of proposed RHCER in terms of energy usage for the transmission of data packets to BS. The highest gain in energy savings has been computed from LEACH, which is about 31%, and from M-LEACH as 26%. This is because the proposed system distributes the strain of energy use among sensor nodes uniformly. The proposed methodology selects appropriate nodes for cluster placement in agricultural land based on a decision made using multiple criteria function. Furthermore, rather than being scattered, the process of CH selection, cluster formation, and zone distribution is controlled by BS, which significantly reduces the ratio of energy exhaustion in the observing field. Furthermore, the suggested framework avoids the process of recurring re-clustering and re-routing due to the multiple variables as a decision function. Because the most appropriate cluster heads are selected based on energy efficiency, distance to BS, and signal intensity characteristics, the proposed framework considerably reduces energy usage in the agriculture domain.

#### 5.2.3. Packet Drop Ratio

**Figure 4:**
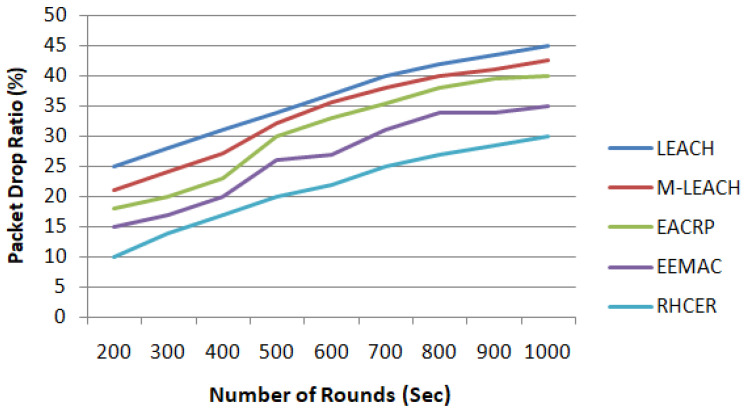
The impact on packet drop ratio with varying number of simulation rounds.

Figure 4 depicts the numerical results of the RHCER in comparison to other alternatives in terms of packet drop ratio. The RHCER significantly improved the packet drop rates compared to earlier solutions. The existing method ignored the importance of the transmission link element, resulting in higher drop rates. Our suggested architecture measures signal intensity as well as the remaining energy of nodes throughout data forwarding, resulting in efficient routing performance. Furthermore, the suggested framework enhances the packet delivery ratio by analyzing the multiple criteria-making process and avoiding crowded data routing nodes. Furthermore, the suggested framework includes the distance to and from the BS factor.

## 6. Conclusions

The nodes in WSN are powered by modest batteries, and the amount of energy available from these sources is restricted. The preservation of energy is an essential issue in the large-scale development of a WSN-assisted IoT network. This research proposes a unique energy-effective routing protocol, RHCER, based on the network subdivision technique, as well as a multi-tier-based clustering architecture. We also devised an approach for region and sub region construction that varies with network area dimensions to suit the need of an energy-efficient, resilient network. The proposed framework has been simulated for a varying number of rounds and performance, and has been analyzed for various performance metrics. Further, to check the effectiveness of our proposed solution, the retrieved results from the proposed solution have been compared against existing clustering protocols. The comparative analysis proved the outstanding performance of RHCER for all the tested parameters in comparison to earlier considered protocols. However, the scheme has limitations in maintaining secure transmission inside network nodes. To work in this direction, this research can be further extended in the future while applying blockchain-based security solutions during content transmission. Moreover, applying artificial intelligent techniques in the future for the prediction of frequently accessed content to be stored inside cluster heads may enhance network performance.

## Figures and Tables

**Table 1:** Comparison with Existing Solutions.

Ref	Protocol	Description & Benefits	Limitations
[17]	LEACH	Considers single-hop communication, formation of cluster heads, general framework suitable to all applications, medium energy consumption	Poor network lifetime, random CH selection, non-scalable
[18]	LEACH-C	Considers centralized single-hop communication network, formation of cluster heads based on node energy level, uniform distribution of clusters, medium energy consumption	BS involvement, non-even load distribution
[19]	M-LEACH	Multi-hop communication, Low energy consumption, self slot allocation, applicable on general applications.	Data are transmitted across numerous intermediary nodes before arriving at the final destination.
[17]	LEACH	Considers single-hop communication, formation of cluster heads, general framework suitable to all applications, medium energy consumption.	Poor network lifetime, random CH selection, non-scalable.
[13]	HEED	Suitable for source-driven as well as data-driven applications, adopts integrated data aggregation, in which every node collects and transmits its own data to the CH either directly or via a parent. Based on each neighbor node’s closeness to the BS, the parent selection module calculates the connection cost for each of them. Data loss and connection symmetry affect how well a communication is done.	Minimal clustering effect.
[20]	EKMT	K-mean clustering algorithm, cluster formation, and network overload are decreased by CH selection depending on the node’s remaining energy with no BS involvement.	Ineffective since it is vulnerable to intentional exploitation and may be damaging to sensor data.
[21]	ICCHR	Chain-based hierarchical framework, low overhead, efficient data aggregation	Complex structure, difficult to operate.
[22]	EACR-LEACH	Identifies its neighbors before forming clusters. The desired value, which is equivalent to the node degrees of CH, is used to set the greatest possible number of cluster members, reduced energy consumption.	The CH selection mechanism takes too many parameters to make decisions.
	Proposed EEDC	Multi-tier hierarchical clustering framework, even load distribution, subdivision technique.	

**Table 2:** Evaluation parameters with values.

Parameter	Value
Network Area	100 × 70 m${}^{2}$
No.of Zones	4
Initial Energy of nodes	5 Joules
Energy Threshold	0.8 Joules
Packet Size	30 bytes
Topology	Static
CH communication range	30 m
Energy depletion for packet transmission	50 pj/bit/m${}^{2}$

## Data Availability

Not applicable.

## References

[1] Akyildiz I.F., Su W., Sankarasubramaniam Y., Cayirci E. (2002). Wireless sensor networks: A survey. Comput. Netw..

[2] Wang N.C., Hsu W.J. (2020). Energy efficient two-tier data dissemination based on Q-learning for wireless sensor networks. IEEE Access.

[3] Cai Z., Chen Q. (2020). Latency-and-coverage aware data aggregation scheduling for multihop battery-free wireless networks. IEEE Trans. Wirel. Commun..

[4] Li H., Wu C., Yu D., Hua Q.S., Lau F.C. (2012). Aggregation latency-energy tradeoff in wireless sensor networks with successive interference cancellation. IEEE Trans. Parallel Distrib. Syst..

[5] Kandris D., Nakas C., Vomvas D., Koulouras G. (2020). Applications of wireless sensor networks: An up-to-date survey. Appl. Syst. Innov..

[6] Ramya R., Brindha T. (2022). A comprehensive review on optimal cluster head selection in WSN-IOT. Adv. Eng. Softw..

[7] Abasıkeleş-Turgut İ., Altan G. (2021). A fully distributed energy-aware multi-level clustering and routing for WSN-based IoT. Trans. Emerg. Telecommun. Technol..

[8] Chen F., Wang A., Zhang Y., Ni Z., Hua J. (2021). Energy efficient SWIPT based mobile edge computing framework for WSN-assisted IoT. Sensors.

[9] Hisham M., Elmogy A., Sarhan A., Sallam A. (2020). Energy efficient scheduling in local area networks. Wirel. Netw..

[10] Liu X. (2012). A survey on clustering routing protocols in wireless sensor networks. Sensors.

[11] An M.K., Cho H., Zhou B., Chen L. Minimum latency aggregation scheduling in internet of things. Proceedings of the 2019 International Conference on Computing, Networking and Communications (ICNC).

[12] Hussain M.Z., Hanapi Z.M. (2023). Efficient Secure Routing Mechanisms for the Low-Powered IoT Network: A Literature Review. Electronics.

[13] Younis O. (2004). HEED: A hybrid, energy-efficient, distributed clustering approach for ad hoc sensor networks. IEEE Trans. Mob. Comput..

[14] Moussa N., Khemiri-Kallel S., El Belrhiti El Alaoui A. (2022). Fog-assisted hierarchical data routing strategy for IoT-enabled WSN: Forest fire detection. Peer Peer Netw. Appl..

[15] Shukla A., Tripathi S. (2020). A multi-tier based clustering framework for scalable and energy efficient WSN-assisted IoT network. Wirel. Netw..

[16] Sankar S., Ramasubbareddy S., Luhach A., Nayyar A., Qureshi B. (2020). CT-RPL: Cluster tree based routing protocol to maximize the lifetime of Internet of Things. Sensors.

[17] Heinzelman W.B., Chandrakasan A.P., Balakrishnan H. (2002). An application-specific protocol architecture for wireless microsensor networks. IEEE Trans. Wirel. Commun..

[18] Shi S., Liu X., Gu X. An energy-efficiency Optimized LEACH-C for wireless sensor networks. Proceedings of the 7th International Conference on Communications and Networking in China.

[19] Mhatre V., Rosenberg C. Homogeneous vs heterogeneous clustered sensor networks: A comparative study. Proceedings of the 2004 IEEE International Conference on Communications (IEEE Cat. No. 04CH37577).

[20] Jain B., Brar G., Malhotra J. (2018). EKMT-k-means clustering algorithmic solution for low energy consumption for wireless sensor networks based on minimum mean distance from base station. Networking Communication and Data Knowledge Engineering.

[21] Vidhya G. (2021). Energy-efficient enhanced hierarchical routing chain based clustering for wireless sensor networks. Turk. J. Comput. Math. Educ. (TURCOMAT).

[22] Sennan S., Alotaibi Y., Pandey D., Alghamdi S. (2022). EACR-LEACH: Energy-Aware Cluster-based Routing Protocol for WSN Based IoT. Comput. Mater. Contin..

[23] Lindsey S., Raghavendra C.S. PEGASIS: Power-efficient gathering in sensor information systems. Proceedings of the IEEE Aerospace Conference.

[24] Jafri M.R., Javaid N., Javaid A., Khan Z.A. (2013). Maximizing the lifetime of multi-chain PEGASIS using sink mobility. arXiv.

[25] Haseeb K., Ud Din I., Almogren A., Islam N. (2020). An energy efficient and secure IoT-based WSN framework: An application to smart agriculture. Sensors.

[26] Begum B.A., Nandury S.V. (2022). A Survey of Data Aggregation Protocols for Energy Conservation in WSN and IoT. Wirel. Commun. Mob. Comput..

[27] Saini P., Sharma A.K. (2010). Energy efficient scheme for clustering protocol prolonging the lifetime of heterogeneous wireless sensor networks. Int. J. Comput. Appl..

[28] Pedditi R.B., Debasis K. (2023). Energy Efficient Routing Protocol for an IoT-Based WSN System to Detect Forest Fires. Appl. Sci..

[29] Rani S., Koundal D. (2021). An optimized framework for WSN routing in the context of industry 4.0. Sensors.

